# White Light Diffraction Phase Microscopy in Imaging of Breast and Colon Tissues

**DOI:** 10.3390/diagnostics14171966

**Published:** 2024-09-06

**Authors:** Adriana Smarandache, Ruxandra A. Pirvulescu, Ionut-Relu Andrei, Andra Dinache, Mihaela Oana Romanitan, Daniel Constantin Branisteanu, Mihail Zemba, Nicoleta Anton, Mihail-Lucian Pascu, Viorel Nastasa

**Affiliations:** 1 Laser Department, National Institute for Laser, Plasma and Radiation Physics, 077125 Magurele, Romania; adriana.smarandache@inflpr.ro (A.S.); ionut.andrei@inflpr.ro (I.-R.A.); andra.dinache@inflpr.ro (A.D.); mihai.pascu@inflpr.ro (M.-L.P.); 2 Department of Ophthalmology, University of Medicine and Pharmacy “Carol Davila”, 020022 Bucharest, Romania; mhl.zmb@yahoo.com; 3 Department for Emergency Internal Medicine and Neurology, Stockholm South General Hospital, 11883 Stockholm, Sweden; mihaela.romanitan@regionstockholm.se; 4 Department of Ophthalmology, University of Medicine and Pharmacy “Grigore T Popa”, 700115 Iasi, Romania; daniel.branisteanu@umfiasi.ro (D.C.B.); anton.nicoleta1@umfiasi.ro (N.A.); 5 Extreme Light Infrastructure-Nuclear Physics ELI-NP, “Horia Hulubei” National Institute for Physics and Nuclear Engineering IFIN-HH, 077125 Magurele, Romania; viorel.nastasa@eli-np.ro

**Keywords:** breast tissues, colon tissues, quantitative phase imaging, refractive index, tumor tissue, white light diffraction phase microscopy

## Abstract

This paper reports results obtained using white light diffraction phase microscopy (wDPM) on captured images of breast and colon tissue samples, marking a contribution to the advancement in biomedical imaging. Unlike conventional brightfield microscopy, wDPM offers the capability to capture intricate details of biological specimens with enhanced clarity and precision. It combines high resolution, enhanced contrast, and quantitative capabilities with non-invasive, label-free imaging. These features make it a useful tool for tissue imaging, providing detailed and accurate insights into tissue structure and dynamics without compromising the integrity of the samples. Our findings underscore the potential of quantitative phase imaging in histopathology, in the context of automating the process of tissue analysis and diagnosis. Of particular note are the insights gained from the reconstructed phase images, which provide physical data regarding peripheral glandular cell membranes. These observations serve to focus attention on pathologies involving the basal membrane, such as early invasive carcinoma. Through our analysis, we aim to contribute to catalyzing further advancements in tissue (breast and colon) imaging.

## 1. Introduction

Breast and colon cancer collectively accounted for about 21% of new cancer cases in 2023, according to GLOBOCAN [1], making them among the most prevalent malignancies worldwide. This underscores the urgent need for novel strategies aimed at early detection and treatment, given the significant impact of disease progression on patient outcomes, including cure and survival rates. A notable trend in pathological anatomy involves the development of innovative methods capable of providing rapid and precise interpretation of biopsy samples.

Recent research combines holography and microscopy to perform highly sensitive measurements that can detect very small refractive index (RI) variations in thin biological specimens [2,3,4].

White light diffraction phase microscopy (wDPM), a specific quantitative phase imaging (QPI) technique, has been widely used as a reliable and high-throughput method for reconstructing the complex image field associated with an object. This is achieved by recording the phase shift of light as it passes through various refractive media within the object [5,6]. The common-path design structure of wDPM provides significant advantages, ensuring phase stability against noise and allowing high temporal phase sensitivity. Its off-axis geometry, combined with the temporal and spatial sensitivity inherent to white light illumination in wDPM, closely influences its performance [7]. As a diagnostic tool, wDPM holds promise due to its affordability and ease of implementation compared to other label-free imaging methods. Its stable and reproducible nature, stemming from its common-path and single-shot interferometric technique, facilitates high-throughput acquisition. Moreover, wDPM systems, utilizing white light illumination, offer good spatial phase sensitivity and minimal speckle noise [5].

White light diffraction phase microscopy has emerged as a valuable tool for studying thin samples like cells [7,8,9,10], thanks to its ability to provide detailed imaging with relative ease. However, its application to thicker samples such as tissues has been less explored due to limitations in its space–bandwidth product compared to phase shifting approaches. Off-axis techniques (such as DPM) offer single-shot, high time-bandwidth phase measurements, but at the cost of either spatial resolution or field of view due to the ultimate resolution being determined by the interferogram’s period rather than just the diffraction limit [7].

wDPM has been used in a study of cell division in *E. coli* [11], to monitor the erythrocyte surfaces in patients with epilepsy [12], to produce the first imaging of living red blood cells (RBC) [13], and to image cell structure components, such as nucleoli of HeLa cells (ATCC, CCL-2) [8]. Currently under-represented in tissue imaging, wDPM has been recently used to obtain phase contrast images of ophthalmic tissues samples [14]. Also, Shan et al. [7] published data about the analysis of images of prostate tissues using wDPM.

Other QPI approaches like spatial light interference microscopy (SLIM) [15,16], white light Fourier phase microscopy (wFPM) [17], nanoNAM [18], and digital holographic microscopy (DHM) [19] have been employed to provide quantitative optical pathlength (OPL) maps associated with transparent specimens, including unstained tissue sample biopsies.

This study seeks to examine tissue sample images obtained using wDPM for pathological analysis of both malignant and non-malignant breast and colon tissue specimens. These samples, whether unstained or colored with standard dyes, were analyzed by comparing them with corresponding regions of interest (ROIs) selected from images captured via conventional optical microscopy. Such an investigation holds the potential to stimulate further exploration in tissue pathology utilizing wDPM. Moreover, recent advancements in artificial intelligence (AI) are poised to pave the way for novel applications, facilitating the systematic and comprehensive utilization of data obtained through wDPM [20].

## 2. Description of wDPM

wDPM is an advanced imaging technique that leverages the interference patterns generated by light as it passes through a specimen, capturing phase shifts that correspond to variations in the optical path length. This allows for high-resolution imaging of transparent specimens, such as biological tissues, without the need for labels or stains [14,15,16,17,21,22,23,24].

White-light diffraction phase microscopy (wDPM) offers a significant advancement in imaging by enabling single-shot capture of high-resolution images with both spatial and temporal sensitivity. wDPM can be integrated as an add-on module to a standard commercial microscope (Axio Observer 7, Zeiss, Oberkochen, Germany) without the need for specialized phase contrast optics. The setup utilizes spatially coherent white light illumination from an LED lamp, commonly found in commercial microscopes, and achieves this by minimizing the condenser aperture (NA = 0.09) to ensure spatial coherence across the entire field of view.

In the wDPM setup, a diffraction grating placed at the image plane of the microscope generates multiple diffraction orders, each containing complete spatial information about the sample. The system isolates the zeroth and first diffraction orders at the Fourier plane using a spatial filter. The zeroth-order beam is low-pass filtered to retain only the DC component, while the first-order beam, which serves as the imaging field, is fully transmitted. These beams interfere to create a spatially modulated image, which is captured by a CMOS camera (Zylla 4.2 from Belfast, Northern Ireland).

One of the key strengths of wDPM is its common-path geometry, which ensures that the optical path lengths for the sample and reference beams are identical. This design makes the system inherently stable, minimizing sensitivity to wavelength variations and the temporal coherence of the illumination source. The quantitative phase image of the sample is extracted from the captured interference pattern using a spatial Hilbert transform, allowing for precise measurement of phase shifts caused by the sample. This capability is particularly valuable for imaging transparent, unstained biological specimens, where traditional brightfield microscopy offers limited contrast.

In terms of technical specifications, the wDPM system used in this paper includes a diffraction grating with a period of 9.09 μm (110 lines/mm), optimized to be smaller than the diffraction-limited spot of the microscope’s imaging system. The system’s lens configuration (L1–L2) provides additional magnification (2.5×), ensuring that the sinusoidal modulation of the image is adequately sampled by the CCD camera. The achromatic lenses used throughout the setup minimize chromatic dispersion, further enhancing image clarity.

A key aspect of wDPM is its handling of the space–bandwidth product (SBP), which defines the balance between spatial resolution and field of view. While wDPM offers high-resolution imaging, the SBP inherently limits the maximum area that can be imaged at this resolution. This trade-off is crucial in applications where both detailed resolution and a large field of view are desired. However, wDPM mitigates some of these limitations by employing spatiotemporal frequency bandpass filtering, which enhances the system’s pathlength sensitivity toward the picometer scale at realistic bandwidths. This capability allows for precise detection of subtle phase shifts, even within the constraints of the SBP.

Moreover, the wDPM design used in this paper, which includes a common-path geometry and a robust lens configuration, ensures that the system remains stable and consistent, minimizing the impact of SBP limitations on imaging performance. The system’s achromatic lenses reduce chromatic dispersion, further optimizing image quality and resolution.

In summary, while wDPM is subject to the space–bandwidth product limitations common to high-resolution imaging techniques, its combination of spatial and temporal sensitivity, along with innovative filtering strategies, pushes the boundaries of what can be achieved within these constraints. This makes wDPM a powerful tool for detailed and sensitive imaging in biomedical applications [8].

## 3. Materials and Methods

Several breast and colon tissue biopsy pieces were surgically harvested while maintaining safety margins.

Specifically, we examined a total of 4 breast tissue samples, with 2 diagnosed as ductal hyperplasia, 1 diagnosed as invasive breast carcinoma, and 1 as a healthy control sample. For colon tissues, 4 biopsy samples were analyzed, including 2 samples diagnosed with malignant colon carcinoma and 2 healthy controls. Out of these samples, more than 100 regions of interest were selected and analyzed.

As a first step, a histopathologic examination was performed on them. All patients from whom samples were collected and used for this study signed an informed consent, in accordance with the Declaration of Helsinki [25]. The biopsy material was processed in several stages according to the standard protocol: fixation, dehydration, clearing, wax infiltration, embedding, sectioning, and staining [21]. Slices of 3–4 µm thickness of unstained breast samples, as well as colon fragments marked with dye hematoxylin and eosin (H&E), were first analyzed by conventional optical microscopy using an inverted microscope (Axio Observer 7, Zeiss, Oberkochen, Germany) equipped with a 20× objective (0.4 numerical aperture—NA). Images of the same samples were subsequently taken by wDPM, using a setup that is similar to the one described in detail by Kim et al. [9].

The spatially resolved quantitative phase image associated with the sample is retrieved from a single camera recording through a spatial Hilbert transform, as described in [13].

**Figure 1:**
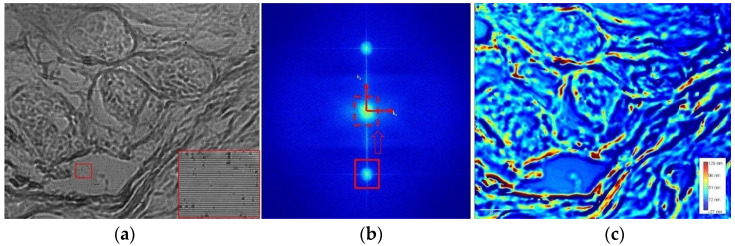
The phase reconstruction procedure. (**a**) The raw image of an unstained breast specimen obtained because of the interference between the first and zero diffraction orders. The enlarged area in red shows the interference fringes. (**b**) Fourier transform of the raw image. (**c**) Quantitative phase map reconstructed after applying the inverse Fourier transform and extracting the background. Color bar represents the optical pathlength values expressed in nanometers.

The fast phase reconstruction algorithm is illustrated in Figure 1. It is based on NVIDIA’s CUDA programming model and uses a calibration background image, which is subsequently subtracted to generate a high-contrast image of the specimen, free of instrument imperfections and specimen-unrelated light patterns.

A Fourier transform is applied to the raw interference image of the sample, generating its power spectrum. One of the diffraction orders obtained in the power spectrum is selected and moved to the center of the image frequency spectrum to obtain the phase map of the sample. An inverse Fourier transform is then applied to bring it back into the space domain, thus generating a phase map of the specimen [22,23]. The background noise of the wDPM was evaluated in [26] by computing a histogram of spatiotemporal OPL noise. This calculation yielded a standard deviation of 1.13 nm for its distribution.

## 4. Results and Discussion

The selected samples were studied with brightfield (BF) microscopy, as well as wDPM, using a 20×/0.4 NA objective installed on the inverted microscope. Quantitative information can be obtained using wDPM, as the phase map corresponds to differences in optical path lengths caused by slight variations in the refractive index within the samples.

The following types of tissues were processed:

(a) Breast tissue with benign lesions (typical ductal hyperplasia, unstained specimens);
(b) Colon tumor tissue; standard H&E—stained samples.
(c) Normal colon tissue; standard H&E—stained samples.

Glandular structures, blood vessels, blood line elements (red blood cells—RBCs, lymphocytes, plasma cells, and neutrophilic polymorphonuclear (PMN) cells), and stromal connective constituents were all examined in normal and harmed tissues.

The following cell types were identified in the phase map based on their characteristics expressed at the three components (nucleus, cytoplasm, and cell membrane): *glandular cells in colon tumors (adenocarcinoma); *glandular cells in normal colon; *endothelial cells; *fibrocyte cells; *RBC; and *lymphocytes/plasma cells/PMNs.

Thin biological samples have weak absorption/scattering of light, and therefore, are practically transparent in white light. The resolution of a microscope operating in transmission mode can be calculated using Abbe’s formula [5,24]:

Δρ = 1.22λ/(NA_O_ + NA_C_) (1)

where Δρ represents the diffraction spot radius or the distance from the peak to the first zero of the Airy pattern; λ is the light wavelength; NA_O_ is the numerical aperture of the objective, while NA_C_ represents the numerical aperture of the condenser. The spatial resolution of our wDPM system, calculated according to the Rayleigh criterion, is approximately 1.35 µm.

The image contrast is produced in QPI techniques by varying the OPL across the sample [27]. The resulting image is a phase map ϕ(*x*, *y*) that is a quantitative measure of the product of the tissue’s thickness (t) and the difference between the tissue’s RI, n_t_, and that of its adjacent medium, n_m_. This relationship is given by

(2) $$\phi \left(x,y\right)=t(x,y)\left[n_{t}\left(x,y\right)-n_{m}(x,y)\right]\frac{2\pi }{\lambda }$$

where 2*π/λ = k*, represents the incident wave vector and *λ* is the wavelength of light. Diagnosis based on QPI thus provides the potential to remove inter- and intra-observer variations, considering that the information found in the phase map $\phi \left(x,y\right)$ is a quantitative measure of the biopsy tissue morphology [15].

The quantitative analysis of the tissue sample’s morphology is expressed by the OPL (measured in nm) across the sample and is shown in color bars in the wDPM images of the figures.

### 4.1. Breast Tissues Comparative Analysis

**Figure 2:**
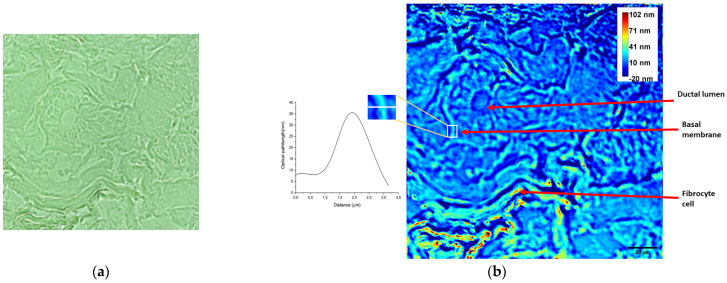
Unstained breast tissue characterized by typical ductal hyperplasia showing a mammary duct cross-section. (**a**) BF image taken with the inverted microscope (20×/0.4 NA objective); (**b**) phase map attained using the wDPM system with the OPL profile plot at the line across the selected region of the basal membrane shown in detail. The color bar represents the OPL through the specimen, in nm (many pixels are saturated at 102 nm in order to enhance details in the ROI); the arrows indicate the ductal lumen, the ductal basal membrane, and a fibrocyte cell.

**Figure 3:**
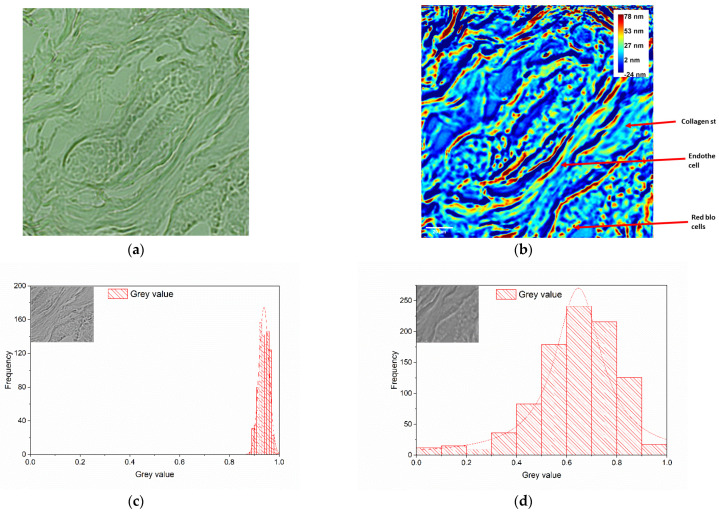
Unstained breast tissue characterized by typical ductal hyperplasia showing mammary ducts in longitudinal section. (**a**) BF image taken with the inverted microscope (20×/0.4 NA objective); (**b**) phase map attained using the wDPM system, where color bar represents the OPL through the specimens, in nm (pixels are saturated at 78 nm in order to enhance details in the ROI); the arrows indicate the collagen stroma, an endothelial cell, and RBCs. Histograms of the collected signal (gray values) on the collagen stroma area are represented for images taken with the inverted microscope (**c**) and wDPM setup (**d**), respectively.

The analysis of the breast tissue samples acquired via conventional microscopy, shown in Figure 2a and Figure 3a, reveals ductal structures with slightly dilated ducts, cylindrical-cubic cells arranged in a three-layered pattern, and periductal connective tissue containing a few blood vessels. A cross-section of a breast duct represents the region of interest (ROI) in Figure 2, while Figure 3 depicts a transverse section through the mammary ducts.

When comparing the same ROI, the wDPM image in Figure 2b shows the ductal lumen with lower OPL, and the duct’s basal membrane appears as a linear structure with slightly variable thickness. The basal membranes of the cylindrical-cubic glandular cells are represented with focal linear and oval-trapezoidal shapes, featuring a central area surrounded by a peripheral extent.

The endothelial cell highlighted in Figure 3b has an elongated shape, with a high RI (large OPL) nucleus in the central area surrounded by distinct regions towards the periphery. The cytoplasm, present in small quantities, appears distinct from the nucleus. The fibrocyte cells have nuclei that transition through several distinct regions from the central area outward, with abundant cytoplasm. Additionally, the collagen bands around the fibrocytes appear darker, longer, and thicker compared to the nuclei of the fibrocytes. Their dimensions are 2–3 times larger than those of the endothelial cells, and they also have an elongated shape. The inflammatory cells (lymphocytes, plasma cells, PMN) maintain a round shape, with their nuclei displaying a small central, a middle, and a peripheral area.

Comparing the histograms of the collagen stroma area from images taken with both the inverted microscope (Figure 3c) and the wDPM setup (Figure 3d) reveals a wider spectrum of values for the wDPM images. This may be due to the better in-depth resolution of wDPM-visualized samples, a finding that could be useful for further automated analysis of histological data, such as in-depth studies of deviations in collagen fibers in breast cancer tissue [28].

### 4.2. Colon Tissues Comparative Analysis

**Figure 4:**
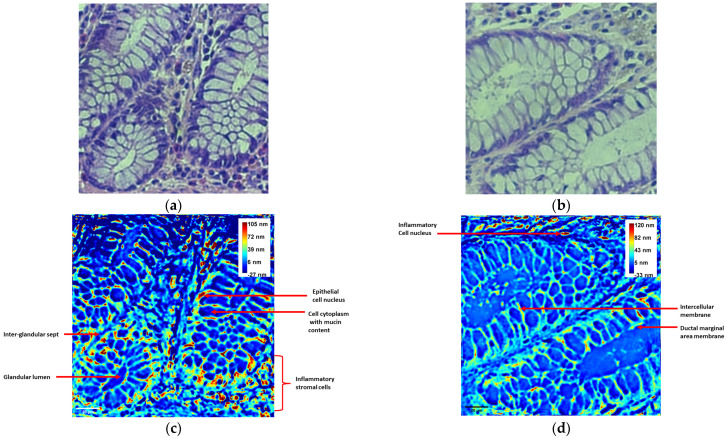
Normal colon tissue—H&E staining. (**a**,**b**) BF images taken with the inverted microscope (20×/0.4 NA objective); (**c**,**d**) phase maps attained using wDPM system, where color bar represents the OPL through the specimens, in nm (pixels are saturated at 105 nm in (**c**), and at 120 nm in (**d**) in order to enhance details in the ROIs).

Both conventional microscopy and wDPM were used to analyze samples of normal colon tissues as well as specimens of cancer-like abnormalities in colon tissues stained with standard H&E. The microscopic anatomopathological analysis of normal colon tissues evidenced glandular structures with regular lumens, delimited by cylindrical cells with basal nuclei and apical cytoplasm, as well as inter-glandular stromal tissue with rare inflammatory elements, as depicted in Figure 4a,b.

In contrast, the wDPM images depicted in Figure 4c,d emphasize the tissue characteristics based on the individual optical path lengths of its components. The cell membrane appears distinctly outlined, exhibiting shades of brown (central), red (middle area), and yellow (peripheral area). The cytoplasm presents a pale blue hue, while the glandular lumen appears deep blue. Inflammatory, endothelial, and fibrocyte cells exhibit similar characteristics to those described for breast tissue samples in Section 4.1.

The cell membranes’ optical thicknesses appear linearly steady, having an oval shape in patches, being brown in the central area, red in the middle area, and yellow in the peripheral area in glandular cells; ductal and peripheral glandular membranes appear more intensely colored, with an oval shape. The cytoplasm and nuclei of glandular cells are shown in pale blue. The glandular lumen appears to have very low RI differences.

The vascular endothelial cells and the fibrocyte cells do not show a stained membrane, but the cytoplasm is colored in pale blue and the nucleus is brown (central), red (middle area), and yellow (peripheral), with an elongated area. Inflammatory cells and RBCs are brown (central), red (middle zone), and yellow (peripheral); with a round shape and smaller size than the glandular cells.

**Figure 5:**
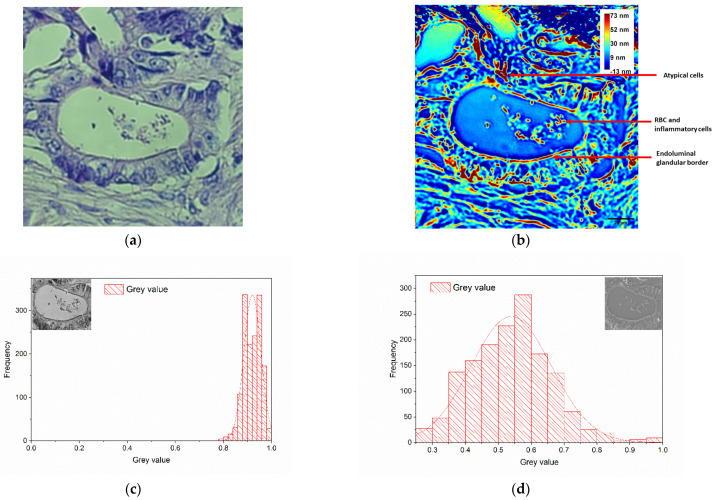
Malignant colon tissue—H&E staining. (**a**) BF image taken with the inverted microscope (20×/0.4 NA objective); (**b**) phase map attained using the wDPM system, where color bar represents the optical pathlength through the specimen, in nm (pixels are saturated at 73 nm in order to enhance details in the ROI). Histograms of the collected signal (gray values) in the selected area are represented for images taken with the inverted microscope (**c**) and wDPM setup (**d**), respectively.

The colon tumor tissue is characterized by malignant glandular structures surrounded by connective tissue, with red blood cells and inflammatory cells in the lumen of the glands, as shown in Figure 5a. The wDPM image in Figure 5b shows that the endoluminal glandular border is colored depending on the optical thickness, with brown (central), red (middle area), and yellow (peripheral area), or only with red and yellow, having a linear appearance. Glandular boundaries are more difficult to observe in the tumor sample images than in non-malignant ones.

In Figure 5b, the peripheral glandular border is represented as in normal colon tissue, but blurred, with an attenuated, segmented linear appearance. The membranes of atypical cells (oval shape) are colored focally, with variable intensity, in brown, red, and yellow. Their cytoplasm and nucleus are both pale blue. Vascular and fibrocyte endothelial cells appear the same as in the breast tissue presented in Section 4.1.

The RBCs are also visible, presenting a round shape. Inflammatory cells (lymphocytes, plasma cells, PMN) appear colored with a small central brown area, red in the middle area, and yellow at the periphery, keeping the round shape. They can be found in the glandular lumen, which has a very low optical thickness.

Using the images obtained with the wDPM setup and the inverted microscope, we analyzed the histogram of the collected signal (gray values). An example of the normalized signal distribution for the two types of images is presented in Figure 5c,d. The results suggest that the phase images have a broader spectrum of values that can be linked to a better in-depth resolution of the sample. This can be useful for further histological automated analysis as it can provide a better signal-to-noise ratio.

The following general observations can be made regarding the appearance of the tissues analyzed based on wDPM images: the cell membranes are depicted as continuous lines, a segment, or a discontinuous oval, colored brown (central), red in the middle area, and yellow (peripheral area) in glandular cells. These are reflected in the highest RIs starting focally, with decreasing values towards the edges.

Ductal and peripheral glandular membranes appear more intensely expressed, equivalent to a higher OPL, and have a linear or oval shape. The cytoplasm and nucleus of glandular cells appear pale blue, while the glandular lumen appears intense blue due to reduced optical thicknesses, corresponding to a low or even negative OPL.

The membranes of the vascular endothelial cells and the fibrocyte cells are not marked, but the cytoplasm is labeled as pale blue, corresponding to low values of optical pathlength. The nucleus is focal brown, surrounded by a red region, and yellow at the periphery, with an elongated area. This is also correlated with the highest RI values in the center, which decrease towards the periphery. Glandular boundaries are more difficult to observe in the tumor than in non-malignant tissues.

Inflammatory cells and RBCs turn brown (central), red (middle zone), and yellow (periphery), also reflected in the highest RIs starting focally, with decreasing values towards the edges. They have round shapes and are smaller than glandular cells.

An important finding is that the wDPM images provide more details on peripheral glandular cell membranes. This result can be used to focus research on lesions in which the basal membrane plays an important role, such as early invasive carcinoma [29,30,31,32].

The physical interpretation of the data, based on scattering-phase theorem, evidences that the attenuation due to the light scattering caused by it passing through an inhomogeneous medium is stronger as the tissue roughness becomes more pronounced. As a result, a thin tissue slice functions as a phase grating, with a diffraction efficiency regulated by the scattering mean free path and an average diffraction angle imposed by the anisotropy factor value [33].

Raw biological samples are often optically transparent in optical microscopy. This results in low-contrast images when viewed through a conventional microscope, as shown in Figure 2a and Figure 3a, as examples. On the other hand, wDPM has been proven to be able to highlight tissue structures and features that are poorly represented in BF, which is an absorption-based contrast method. This is because, even if the sample does not absorb light, it shows an RI distribution that perturbs the wave front. Such an occurrence can be exploited as a source of contrast on its own [34]. The data retrieved from phase maps are important for understanding cell structure and function. The RI of a cell, for example, could reveal its total protein concentration [35], as well as the organization and distribution of subcellular organelles, which are specific to certain biological phenotypes [36]. As a result, the phase signature of a cell type is made up of a collection of quantitative parameters from optical phase maps, which provide information beyond the cell shape. In the circumstance of this study, an improved highlighting of collagen fibers can be seen when the images taken with the wDPM system are analyzed, as shown in Figure 3. Recent papers conclude that collagen within the tissue stroma represents a valuable target in cancer biology. Beyond its structural role, collagen is presumed to also play a role in skin inflammation, cell migration, and differentiation in physiologic and pathologic states [37,38]. Label-free molecular profiling, imaging, and analysis of connective tissues, particularly collagen fibers, have recently been discovered to be of great importance in cancer biology for detecting subtle biochemical changes during tumor progression and perhaps during cancer treatment, particularly in the case of breast malignancy [28,30,39]. The value of collagen as a disease stratification marker is emphasized by Sakakura et al. [40], where the authors demonstrate that label-free analysis of collagen fibers can be performed with high specificity by combining SLIM with deep learning.

While 3–4 µm thick specimens are common for histopathology, multiple scattering is ultimately the process limiting the thickness of tissue measurable by wDPM. We also acknowledge that, since the illumination is spatially coherent (plane wave), the sectioning of wDPM is reduced compared to that of other techniques, which rely on incoherent illumination.

In DPM, one must fulfill the sampling condition both for the sinusoid recorded by the camera, sampled by the pixels, and the diffraction spot, sampled by the sinusoid. As such, the space–bandwidth product is at least 3× smaller than in the case of phase-shifting methods. Recently, it has been shown that shifting the grating in DPM provides a space–bandwidth benefit [7]. Fourier ptychography [41] provides a solution for boosting the space–bandwidth product by using a series of off-axis illuminations and mosaicking the spatial frequency domain. However, this approach would not be practical in a DPM geometry due to the pinhole geometry that must match the illumination.

For histopathology applications, we envision wDPM operation in combination with a computer-controlled scanning stage, which removes any limitation to the field of view. The acquisition speed of such a whole-slide scanner should match that of a brightfield instrument since wDPM is a single-shot method.

## 5. Conclusions and Outlook

This report presents an investigation of some breast and colon tissue samples using white light diffraction phase microscopy (wDPM), which proves to be a promising approach in the field. The study shows a quantitative analysis of tissue sample morphology, elucidating optical pathlength variations across the sample, which is an important factor in determining the image contrast. Moreover, the enhanced contrast of wDPM images is highlighted through a comparison of histograms from selected regions of interest in the breast and colon specimen images obtained via conventional microscopy and the wDPM setup. These findings hold implications for further improving diagnostic accuracy and efficiency in clinical pathology. Integrating quantitative phase imaging techniques like wDPM could make the measurement of tissue structure easier and faster. The heightened interferometrically measured contrast facilitates objective morphological quantification, as evidenced by the comparison of microscopic images with those generated by wDPM, encompassing both unstained and stained tissue samples (see Figure 2, Figure 3, Figure 4 and Figure 5).

The wDPM-derived images in this study offer insights into peripheral glandular cell membranes, guiding future research endeavors focused on tumors where the basal membrane plays a pivotal role, such as early invasive carcinoma. Additionally, by enabling the detailed assessment of collagen fiber alignment and orientation, the method could facilitate personalized patient prognosis prediction.

In conclusion, our data show that wDPM offers good perspectives in tissue examination. One key benefit is its high spatial and temporal resolution. This allows detailed visualization of cellular and subcellular structures in tissues, making it possible to observe dynamic processes in real time. The reduced speckle noise due to the use of white light is another advantage. This improvement leads to clearer images and better contrast, which helps in distinguishing slight variations in the optical pathlength. This is particularly useful in complex tissue environments, where such variations can be indicative of different tissue components or pathological states, as shown in the unstained breast specimen in Figure 2. The non-invasive and label-free character makes wDPM a promising tool for the analysis of samples where preserving the native state is important.

wDPM provides accurate quantitative phase measurements, which are important for assessing cellular and tissue structures’ refractive index distributions. This can be especially useful for distinguishing between different tissue types and detecting pathological changes. Another benefit of using wDPM for tissue examination is that it provides phase information by single-shot measurements, thus allowing for much faster acquisition rates. This comes with the compromise of either spatial resolution or field of view, which we envision mitigating by operation of the wDPM setup in combination with a computer-controlled scanning stage.

At the same time, wDPM can be integrated with existing optical setups and combined with other imaging modalities such as confocal microscopy, computer tomography, RMN, etc. This versatility makes it a valuable tool in comprehensive tissue imaging studies such as the ones described in this paper. To make use of these new imaging techniques in medical diagnosis, we need to develop new standards for interpreting the data they provide. Future research could focus on studying more complex tissue samples to build a comprehensive database of physical characteristics. This approach could also help identify early signs of fibrosis in organs, regardless of whether the tissue is benign or malignant. One of the main conclusions at this stage is that the images obtained by wDPM provide information equivalent to high-performance optical microscopy, at the same time opening perspectives for more accurate results. wDPM has the potential to become a powerful tool in histopathology, offering greater sensitivity and accuracy in tissue analysis and diagnosis. By demonstrating the ability of wDPM to detect minute structural changes that BF microscopy may miss, we emphasized its value in improving early cancer detection and overall diagnostic precision.

## Data Availability

The authors can make available the data supporting result, at kind request, addressed to the corresponding author and/or the first authors of the article.
